# NeuroMorph: A Software Toolset for 3D Analysis of Neurite Morphology and Connectivity

**DOI:** 10.3389/fnana.2018.00059

**Published:** 2018-07-23

**Authors:** Anne Jorstad, Jérôme Blanc, Graham Knott

**Affiliations:** Biological Electron Microscopy Facility, Centre of Electron Microscopy, École Polytechnique Fédérale de Lausanne, Lausanne, Switzerland

**Keywords:** neuroimaging software, 3D modeling, data visualization, serial section electron microscopy, cell morphology, neuron, synapse, connectomics

## Abstract

The geometries of axons, dendrites and their synaptic connections provide important information about their functional properties. These can be collected directly from measurements made on serial electron microscopy images. However, manual and automated segmentation methods can also yield large and accurate models of neuronal architecture from which morphometric data can be gathered in 3D space. This technical paper presents a series of software tools, operating in the Blender open source software, for the quantitative analysis of axons and their synaptic connections. These allow the user to annotate serial EM images to generate models of different cellular structures, or to make measurements of models generated in other software. The paper explains how the tools can measure the cross-sectional surface area at regular intervals along the length of an axon, and the amount of contact with other cellular elements in the surrounding neuropil, as well as the density of organelles, such as vesicles and mitochondria, that it contains. Nearest distance measurements, in 3D space, can also be made between any features. This provides many capabilities such as the detection of boutons and the evaluation of different vesicle pool sizes, allowing users to comprehensively describe many aspects of axonal morphology and connectivity.

## 1. Introduction

The development of volume EM imaging methods now provides unprecedented opportunities to understand the detailed morphology and connectivity of neurons (Briggman and Denk, 2006; Kornfeld and Denk, 2018). Geometrical analysis of the imaged structures, however, requires either measuring the required features directly on the serial images, or interacting with 3D models, once they have been extracted after segmentation. Here we present a set of software tools for exploring, annotating and measuring various features of 3D models.

Open source software such as Fiji^1^(Cardona et al., 2012), KNOSSOS^2^(Helmstaedter et al., 2011), Espina^3^(Morales et al., 2011), Reconstruct^4^(Fiala, 2005), and ITK-SNAP^5^(Yushkevich et al., 2006), and proprietary software including Amira^6^, exist for annotating and measuring features on 2D serial images, and constructing 3D models that can be examined visually but generally not manipulated. In contrast, NeuroMorph has been developed to analyze and interact with the models reconstructed from any of these tools directly in a 3D environment. The NeuroMorph tools augment these models, and perform specialized analyses directly on them.

To explain the different functionalities of these tools, we present how they can be used to measure a range of different features of axonal boutons. This includes how the 3D models can be visualized together with the original image stacks, and features such as synapses and vesicles added to the model. We explain how to make volume, surface area, and length measurements on any part of the model, and how a centerline of an axon can be used to show the changing densities of elements such as vesicles along its length. In addition we include tools for measuring the degree to which structures such as boutons are in contact with other cellular features such as dendrites. This is useful for understanding more about axonal function.

The software and detailed instructions, along with a stack of EM images and corresponding scale models of biological structures including the ones shown in this paper, are available from our website^7^ which links to our GitHub page^8^.

The Blender models created and analyzed with these tools are also compatible with the simulation software MCell^9^ that uses the same Blender software via the CellBlender graphical user interface to simulate various aspects of cellular processes (Kerr et al., 2008).

## 2. Meshes

Blender^10^ is a widely used, free, open-source software package developed primarily for 3D computer graphics applications. NeuroMorph is a toolset comprising “addons” that can be integrated into Blender to provide specialized tools for the analysis of 3D models derived from electron microscopy imagery of neurons. However, much of its functionality can be applied to models derived from any source. This paper extends our previous work (Jorstad et al., 2015), which presented an earlier version of the NeuroMorph Measurements tool, described here briefly in section 4.

**Figure 1:**
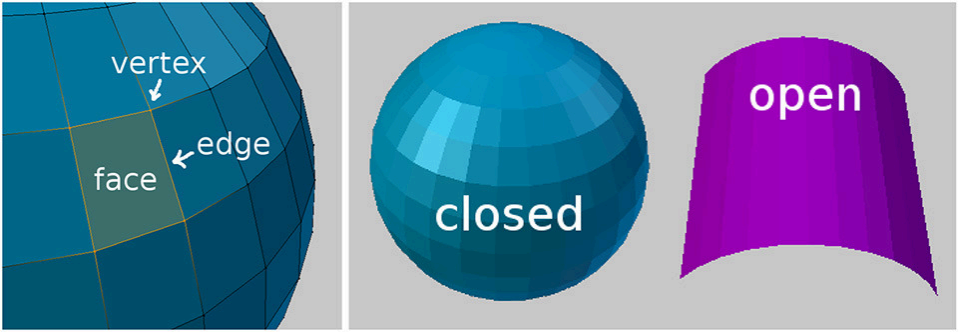
Mesh geometry fundamentals. **(Left)** Part of a sphere, with four orange vertices that are connected by edges, forming a single face. **(Right)** A closed object and an open object.

A 3D model is comprised of a mesh that is defined by points called vertices, edges connecting the vertices, and polygons called faces that are bounded by the edges, to create that 3D surface, see Figure 1. The surfaces can be either closed like a ball or open like a piece of cloth.

Meshes can be loaded into Blender from a variety of sources, or constructed directly within the software itself. For example, .obj files of annotations made in Fiji^11^ can be imported into Blender using the NeuroMorph Import Objects tool (found in the “Other Tools” section of the toolkit).

**Figure 2:**
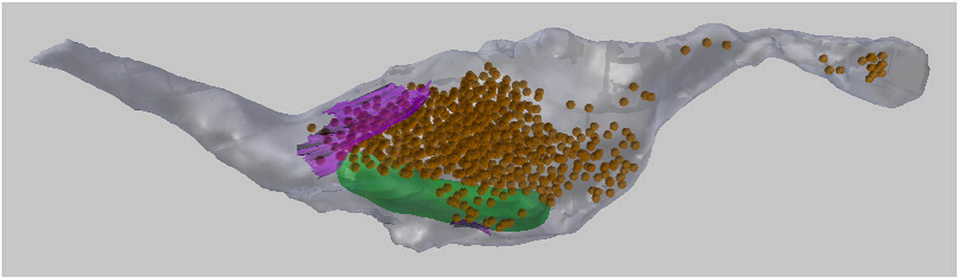
A 3D drawing of a single axon with synaptic bouton reconstructed from serial EM images. The axon (gray) contains synaptic vesicles (orange), synapses (purple), and mitochondria (green). This axon will be used as an example throughout this paper.

In this paper we will analyze the 3D meshes of an axon containing meshes of mitochondria, vesicles, and synaptic contacts (see Figure 2). In section 3 we will describe how to visualize the serial images, as well as how to add spherical meshes at the position of each vesicle, and also create surface meshes representing the synapses. In section 5 we will also make use of a centerline, running through the axon, and consisting of vertices strung together by edges, without any faces. This centerline can be used to carry out various analyses that are useful for describing the geometry of the axon.

## 3. Drawing in 3D

A common method of creating 3D models from serial EM images is to annotate each image pixel by pixel, painting the structure of interest. This can be done in software such as Fiji and Microscopy Image Browser^12^. The models are then exported as 3D objects. This process can be very time-consuming, and the repetitive task of drawing on 2D planes gives little information of the 3D nature of the structure of interest. The NeuroMorph 3D Drawing tool offers a faster alternative, by allowing users to mark and draw onto the serial images directly in the 3D workspace of Blender. The image stack can be efficiently navigated while the 3D structures are being constructed, providing the user with a better sense of the structure as it is being created.

**Figure 3:**
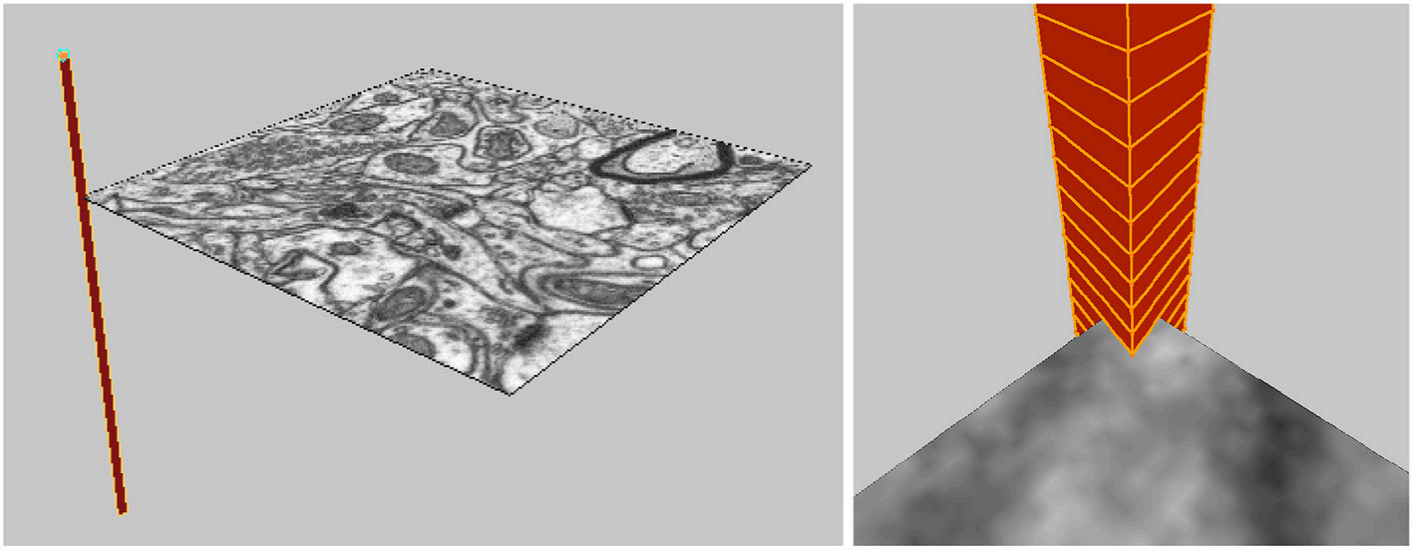
The Image Stack Ladder used for navigation through the 3D image stack.

When the user loads the image stack into the software, an “Image Stack Ladder” object is created in Blender that is the height of the image stack, located in the corner of the images, and consists of small triangular faces pointing to the locations of each image in the stack, see Figure 3. This allows the user to select any vertex in the Image Stack Ladder, and view the image at that location in the image stack. Multiple images can be displayed at once. Clicking on a single image, the user can scroll through the image stack, allowing the user to explore the image stack and see how the 3D objects align with the images.

From a single image stack, with many images stacked in the Z dimension, a tool to generate image stacks of the same data in the X and Y dimensions is also provided, called “Generate 3D Image Stacks” (found in “Other Tools”). All three stacks can be loaded into Blender and explored for extended 3D analysis. Special thanks to Tom Boissonnet for the contribution of this feature.

### 3.1. Plotting synaptic vesicles

**Figure 4:**
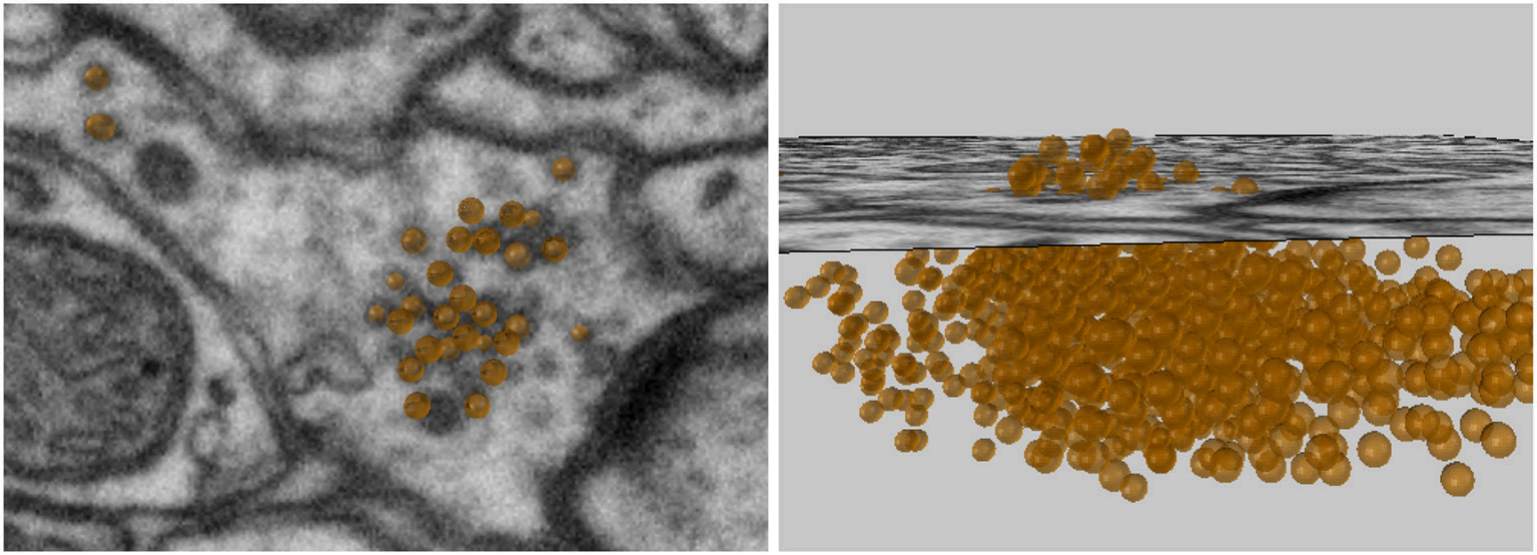
Vesicles spheres annotated with a single click.

The “Mark Points on Image” tool allows the user to create spherical meshes of any size with a single click on an image plane, see Figure 4. Here we use this tool to place spheres of a chosen size in the axonal bouton at the exact position of each vesicle. After placing each vesicle sphere, the user can scroll back and forth between the nearby images to visualize the sphere's placement in 3D. If an annotation is incorrect, it can be quickly deleted and the sphere placed again. In this manner, many vesicles can be quickly plotted. If a large number of spheres are going to be created, an option is available to construct spheres using fewer vertices, which results in meshes that look less smooth, but are computationally more efficient.

The vesicles created with this tool will be used in the Sphere to Surface Distances tool described in section 6 to analyze the proximity of vesicles to the pre-synaptic membrane.

### 3.2. Drawing surfaces

The “Draw Curves and Surfaces on Image” tool allows the user to draw curves on images, connect these curves from several layers of the image stack, and create a 3D surface. Here this is used to construct a pre-synaptic membrane.

**Figure 5:**
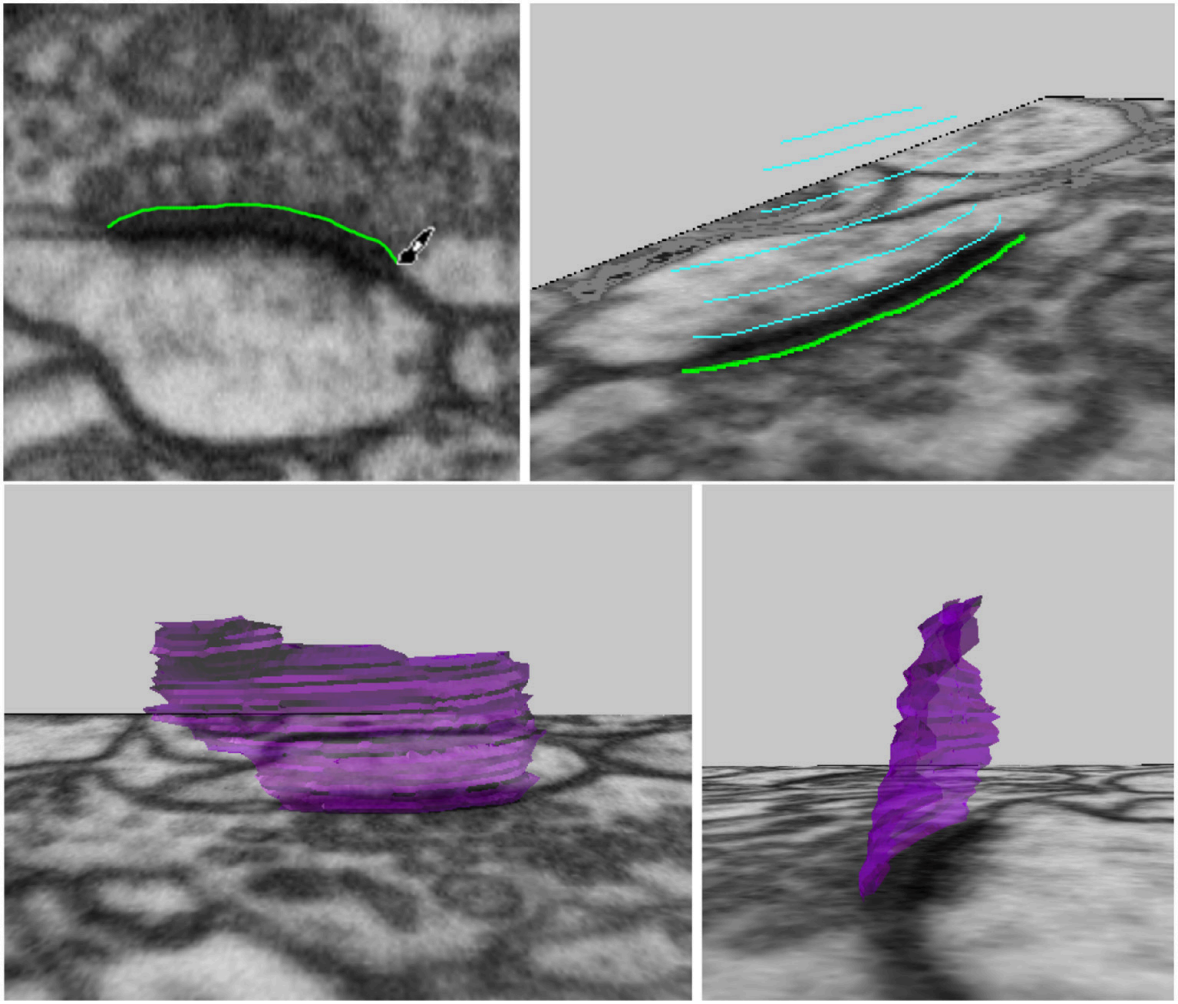
Drawing curves in 3D, and connecting them into a synapse surface.

The user can draw along the boundary of a neuronal structure in an image, erasing if necessary, to correctly outline an object, see Figure 5, top left. When the desired curve has been drawn, it can be converted into a mesh curve consisting of vertices and edges. For faster annotation, the tool can be set to convert curves automatically as soon as the mouse click used for drawing is released. Curves outlining an object should be drawn on several images, either on adjacent images for more precision, or leaving out a few images in between each curve for faster annotation, see Figure 5, top right. Once several curves outlining the same object have been created, the “Construct Mesh Surface from Curves” tool will fit a mesh surface through the curves, adding faces to result in a 3D mesh surface of the object that was outlined, see Figure 5, bottom row.

**Figure 6:**
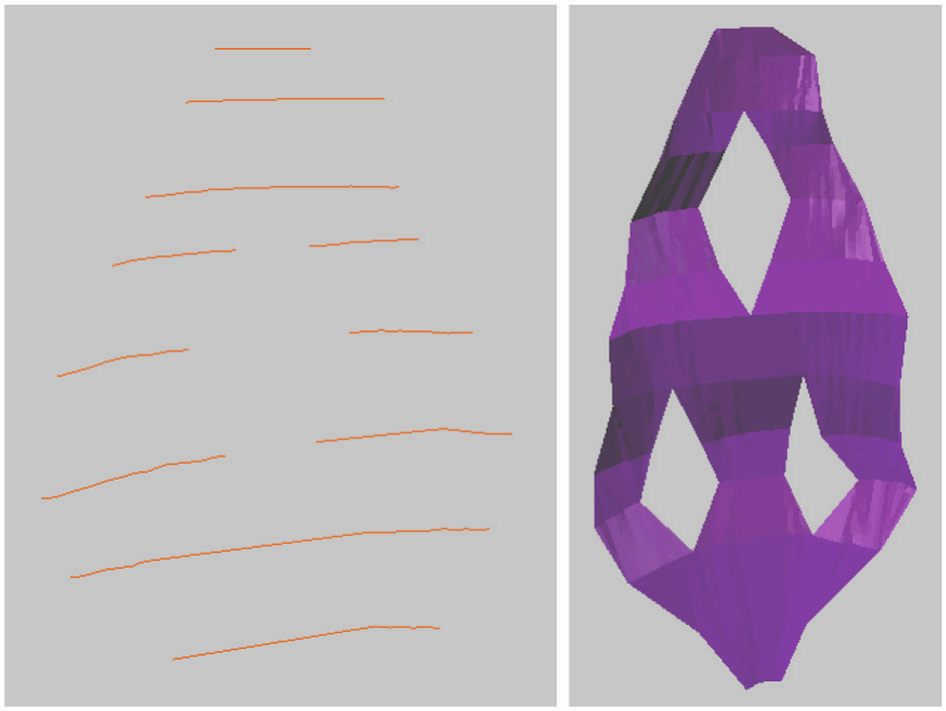
Creating a surface with holes is possible simply by drawing the surface curves on either side of the hole, and leaving the hole empty.

It is also possible to construct surfaces with holes, such as perforated synapses. Simply drawing curves on either side of the hole on each image, with no drawing inside the hole, then constructing the mesh surface from the curves as before, will result in a 3D mesh surface with holes as annotated, see Figure 6.

#### 3.2.1. Limiting cases

When two adjacent curves have very different trajectories, the resulting surface might be incorrect. The algorithm works by fitting a linear surface through each adjacent pair of curves, and then combining all surface segments together at the end to form a single continuous surface. The surface construction functions well when adjacent curves are near parallel, with endpoints not too far from each other. However, when adjacent curves are close to perpendicular, the algorithm cannot be sure which endpoints should correspond, and this can result in a self-intersecting surface. In this case, the user should add more intermediate curves, to better define the progression of the surface being reconstructed.

If a curve is drawn too fast, it is possible that the constructed mesh curve might include extraneous vertices outside of the desired chain of vertices. If this happens, the extra vertices can simply be deleted.

If the constructed surface is not sufficiently smooth for a given application, the user is able to add more intermediate curves to provide as much fine detail as necessary.

### 3.3. Drawing tubes

**Figure 7:**
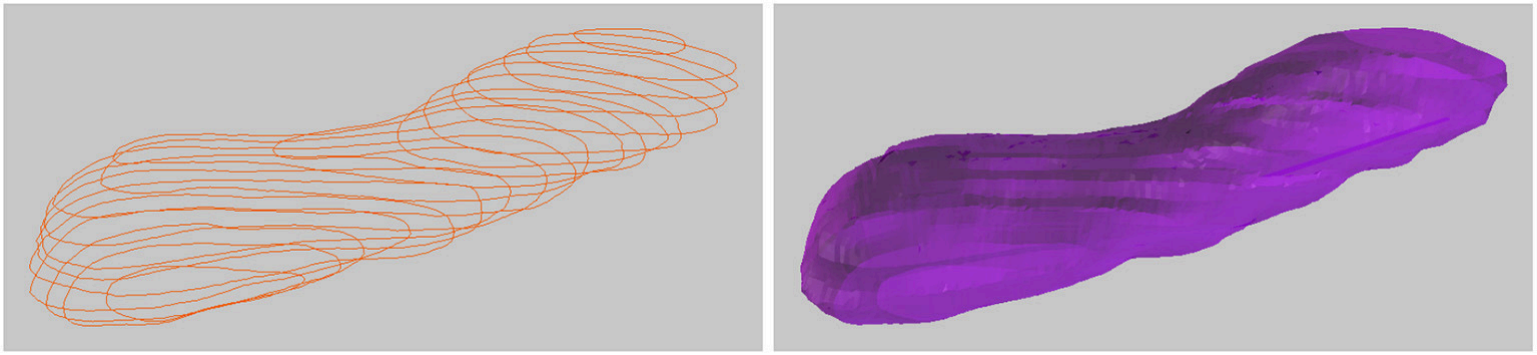
Drawing a closed tubular surface, here a mitochondria.

It is also possible to connect closed curves into closed tubular objects, see Figure 7. Checking “Closed Curve” tells the tool that the curves should be closed, like a circle. Constructing a mesh surface from these closed curves will create a closed tube, and the ends of this tube can also be closed to form a closed object. This tool is not currently capable of handling branching objects, but can be used for annotating tubular objects such as mitochondria. It is possible to handle U-shaped tubular structures with more than one cross section in a single z-plane, but such objects must be constructed in parts with only one cross section per z-plane, and then joined together, which is a simple operation in Blender.

## 4. Measurements

### 4.1. Lengths

The NeuroMorph Measurements tool provides three different length measurement functionalities.

- **Distance Between 2 Points:** calculates the distance between two selected vertices, ignoring the mesh surface.
- **Shortest Distance on Mesh:** calculates the shortest distance between two selected vertices along a path through the vertices of the mesh.
- **Length of Selected Edges:** calculates the total length of all currently selected edges on the mesh.

### 4.2. Surface areas

**Figure 8:**
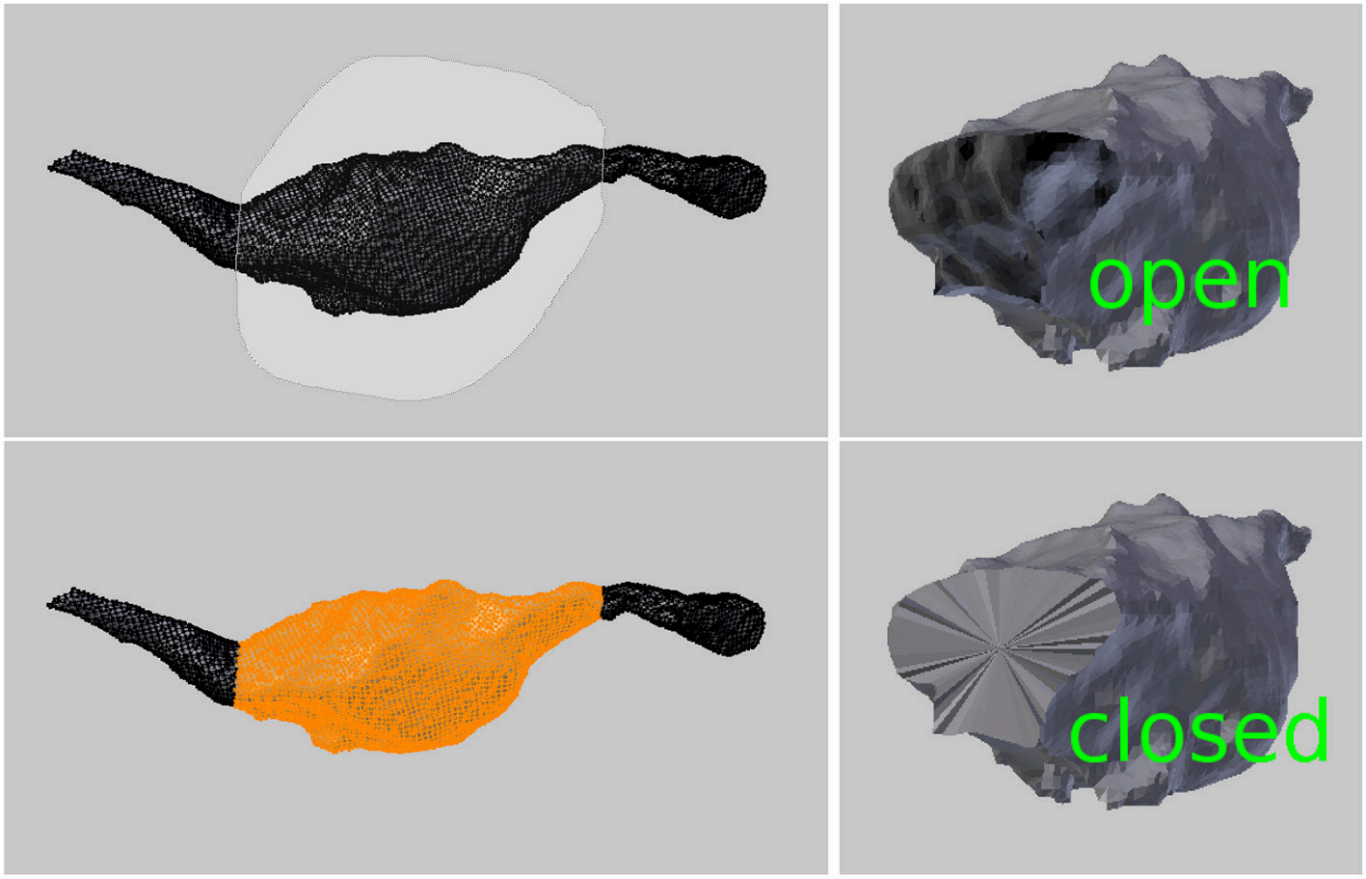
**(Left)** Selecting a region to calculate its surface area and volume. **(Right)** An open surface, and the result after it is closed by the tool to perform the volume calculation.

The surface area of a mesh or any subsection of a mesh can be calculated by highlighting the desired faces and clicking the “Surface Area” button, see Figure 8.

### 4.3. Volumes

The volume of a mesh or any subsection of a mesh can be calculated by highlighting the faces defining the region and clicking the “Volume” button. The tool will first close any holes in the mesh, and then calculate the volume of the closed region, see Figure 8.

For more details on measurement calculations, see Jorstad et al. (2015) where the NeuroMorph Measurements tool and its limitations are described in full.

## 5. Centerline and cross sectional analyses

This suite of tools allows the user to gather structural information about the object (the axon in this case) in terms of the presence of other objects (organelles), or how certain geometric properties change along its length.

The tool first facilitates the creation of a line down the center of the axon, which provides an object along which various properties can be measured. The Vascular Modeling Toolkit (VMTK) (Antiga et al., 2008) is a separate software package that provides a useful tool for centerline creation^13^, based on the algorithm from Antiga and Steinman (2004). The NeuroMorph Centerline tool exports the axon in a format that can be processed by VMTK, and then reads back in the centerline mesh to be used with the rest of this tool. VMTK must be installed separately in order to use this tool.

**Figure 9:**
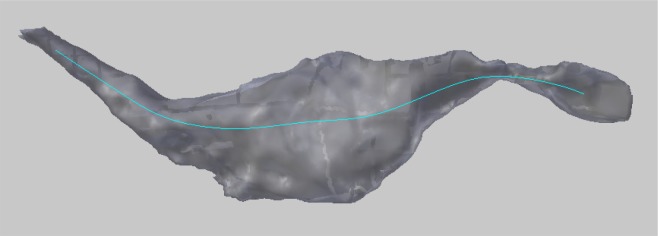
The smooth centerline of an axon.

The constructed centerline does not run down the exact geometric center of the axon, but is instead of smooth curve that always remains on the inside of the structure, and serves as a representative skeleton of the axon, see Figure 9.

Although VMTK is able to construct branching centerlines, the NeuroMorph centerline functionality is based on a single non-branching centerline per axon. To handle branches, a second additional centerline can be constructed, and the calculations performed separately on that branch. Output data can then be combined as determined by the user. U-shaped and S-shaped axons are handed correctly by all the functionality provided by this tool, even in the case when there are multiple cross-section per z-plane.

If the user prefers instead to construct their own axon centerline using standard functionality in Blender, the tools in this section will all function, as long as the centerline is entirely contained inside the axon mesh; extreme precision is not required. The user must simply tell the software about the hand-made centerline by clicking the “Update Centerline” button. The only functionality that is lost by working with user-created or user-edited centerlines is that the minimum axonal radius at each centerline vertex will not be exported. This is data provided by VMTK, and is not re-calculated by NeuroMorph.

The number of vertices that define the centerline can be set by the user. This value determines the precision of the rest of the functions in this section. In practice, we generally use a number of vertices on the order of 200, or a vertex spacing of not less than 100 nm. The points are generally not precisely equally spaced along the centerline curve, but there exact spacing is given in the exported data file.

Functionality is also provided to clean the axon mesh as a pre-processing step before further handling. This removes non-standard geometry such as self-intersections, floating vertices, and other non-manifold geometry that can sometimes result when surfaces are imported from other tools. This mesh cleaning is often necessary in order for other tools, including VMTK, to be able to properly function. Sometimes the input mesh has too many problems, and the tool will delete a large chunk of the mesh (don't worry, every action in Blender can be undone). This is a sign to the user that the mesh should be inspected and modified by hand near the deletion point, possibly by removing some of the problematic regions of the surface and filling in the surface holes with simple faces, an action easily accomplished in Blender. The mesh cleaning function should then be re-run to confirm that the final mesh used for processing is clean. It is recommended to always clean meshes using this tool before they are analyzed, and this functionality has broad utility outside of the context of this suite of tools.

### 5.1. Cross-sectional surface areas

**Figure 10:**
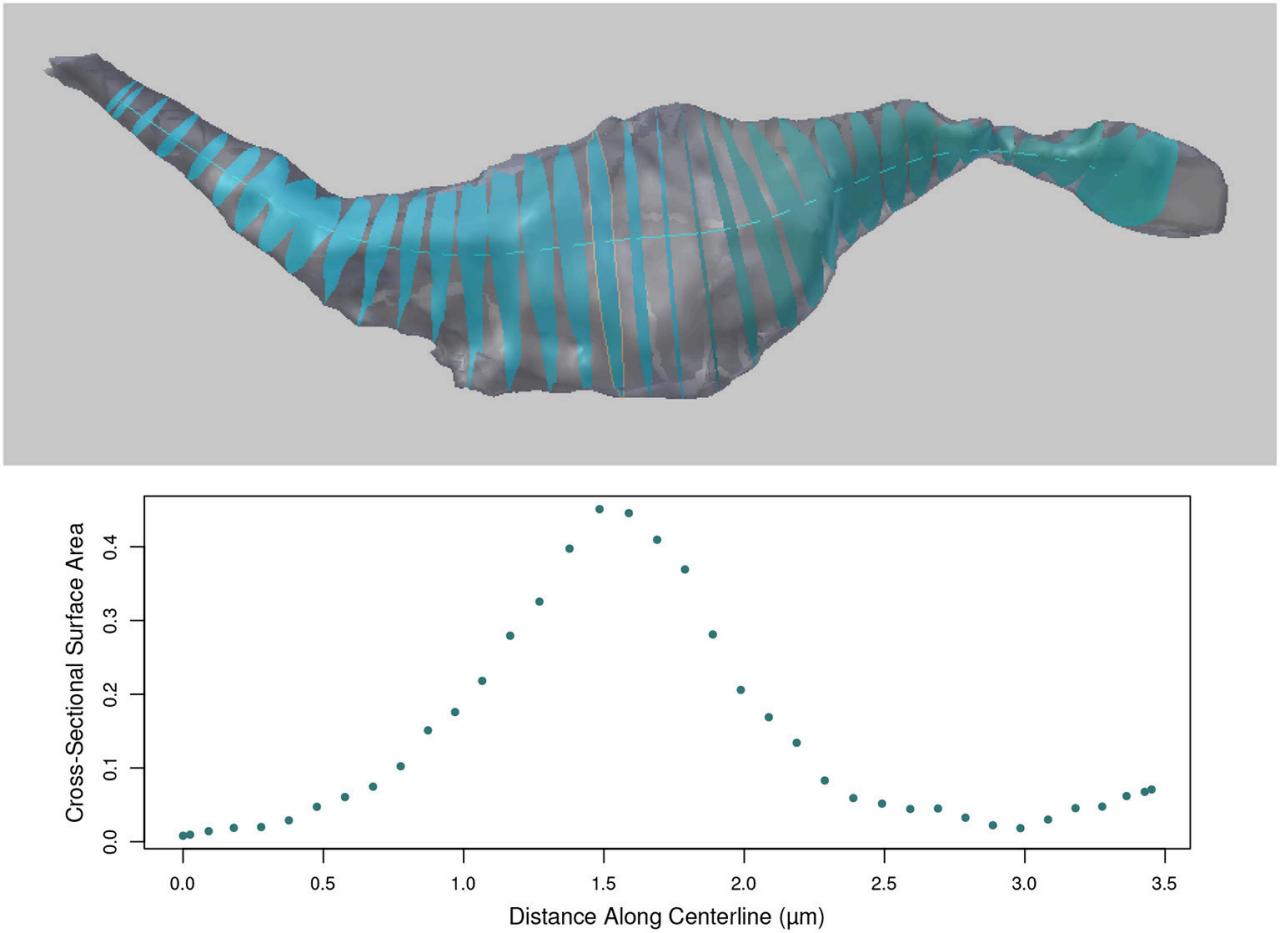
**(Top)** Cross sections of the axon as computed along the centerline. **(Bottom)** The surface areas of the cross sections (in micrometers) at each centerline vertex along the length of the axon.

Once the centerline has been generated, cross-sectional surfaces of the axon can be constructed at every vertex on the centerline, see Figure 10. The cross-sectional surface areas are then calculated along the axon providing a quantitative measure of how the axon's shape changes along its length. This can be used to accurately define the position of axonal boutons, using the bouton detection tool, see section 5.6.

The cross sections are generated perpendicular to the centerline. Therefore, in regions where the centerline is bending back toward itself with high curvature, the user should note that some cross-sections may intersect. However, the area values for each cross section is correct.

#### 5.1.1. Limiting cases

If the diameter of the axon is particularly wide, the tool must be told to use a larger plane when performing the plane-axon intersection calculation that produces the cross section. The computation time of this intersection calculation increases with size, so the tool by default uses a moderately sized plane, but the user is able to adjust the diameter of this intersection plane as necessary via a parameter provided in the user interface.

### 5.2. Max radius of each cross section

This tool calculates the maximum radius of each cross section, as measured from the centroid of the cross section to each of its vertices separately. The centroid is calculated as the average location of all boundary vertices of the cross section, and the intersection point of the centerline with the cross section does not affect this calculation.

Note that for C-shaped cross sections, it is possible that the location of the centroid can technically be outside of the cross section mesh. This does not affect the calculation, and it is up to the user to decide in these cases if the “maximum radius” makes sense.

### 5.3. Project spheres to centerline

**Figure 11:**
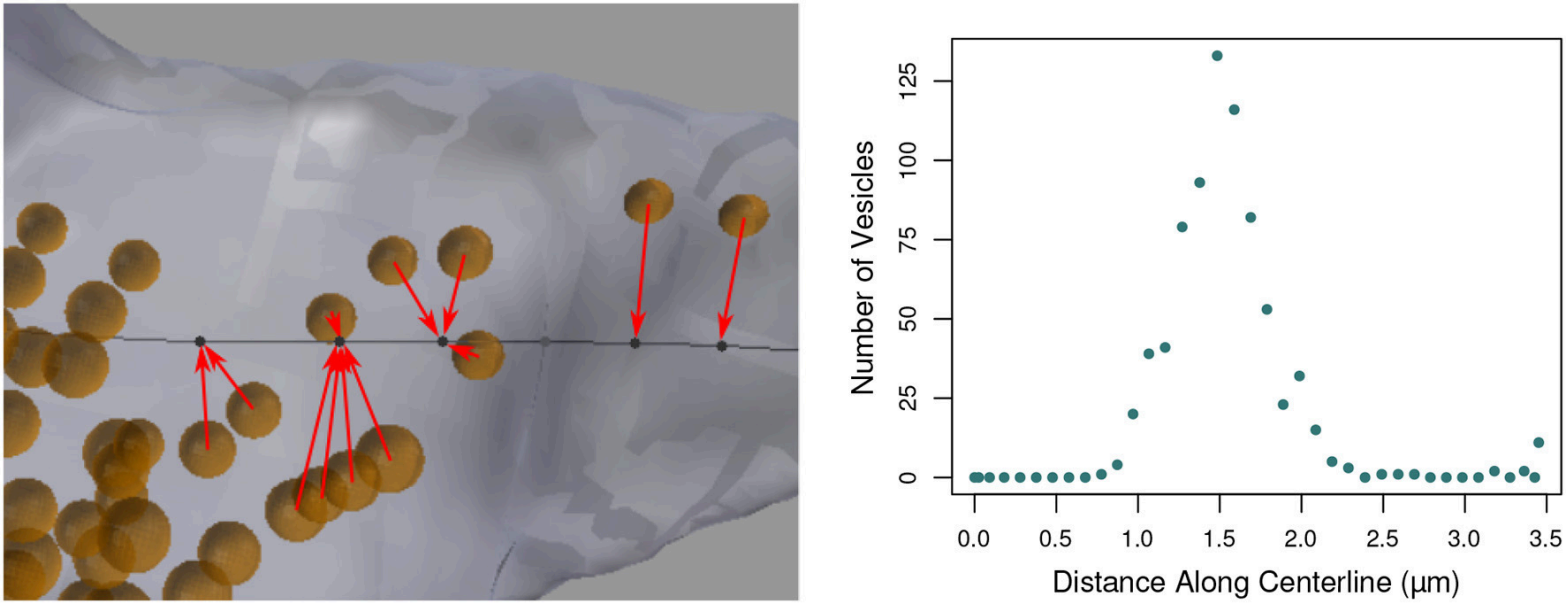
**(Left)** Vesicles (orange) projected to their nearest vertices on the axon centerline (black; for clarity, only a subset of the projection arrows are shown). The tool counts the number of vesicles that project to each centerline vertex. **(Right)** The number of vesicles projected to each centerline vertex along the length of the entire axon, shown in Figure 1.

This function aids in the analysis of the distribution of objects such as vesicles along an axon, see Figure 11. The user provides a collection of input mesh objects, such as the vesicles created in section 3.1, which are assumed to be spheres, but are not required to be. Only the centroid of each object is considered. The centroid of each object is defined as the average (x,y,z) location of its boundary surface vertices, and the user should keep in mind that irregularly shaped objects may not be well-represented by the centroid of their vertices.

The distance from each centroid to each distinct vertex on the centerline is computed, and the object is said to be “projected” to the closest vertex. The number of distinct objects projected to each vertex is tallied, and the function returns the final count of projected objects for each vertex. This data will be exported along with all other data for the centerline.

### 5.4. Project surface areas to centerline

This function aids in the analysis of the distribution of surface contact with objects such as synapses along an axon. The user provides a collection of surfaces which do not have to be continuous.

**Figure 12:**
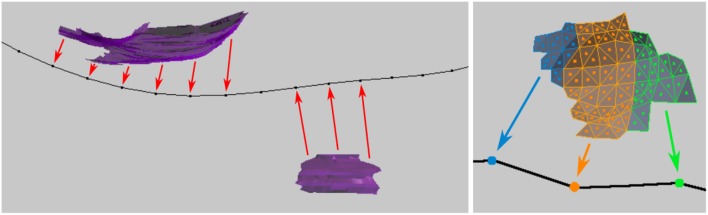
**(Left)** Synapse surfaces (purple) are projected onto the axon centerline vertices. **(Right)** Closeup of the individual faces that make up a surface, each of which is projected to the nearest vertex on the centerline. The tool sums the total surface areas of the faces that project to each vertex.

The distance from the centroid of each surface face to each distinct vertex on the centerline is computed. The individual areas of each surface face projecting to each centerline vertex are summed, and the function returns the total projected area sum for each centerline vertex, see Figure 12. This data will be exported along with all other data for the centerline.

### 5.5. Centerline-based output

Using the tools in this section, a file can be exported for further analysis that contains some or all of the following data for each centerline vertex:

- Length along centerline from endpoint of centerline to this vertex
- Surface area of cross section
- Minimum radius of mesh
- Maximum radius of mesh
- Number of spheres projected
- Sum of chosen surface areas projected

### 5.6. Detect boutons

A final tool is provided that helps the user to define bouton swellings of an axon in a well-defined, reproducible manner, based on certain geometric criteria. Applications of this tool were first reported in Gala et al. (2017).

The user is able to input and experiment with three variables that define the possible beginning and end of a bouton:

$$\begin{array}{l} A=\text{Area Change }(\text{ratio})\\ D=\text{Distance for Area Change}\\ M=\text{Minimum Max Radius} \end{array}$$

**Figure 13:**
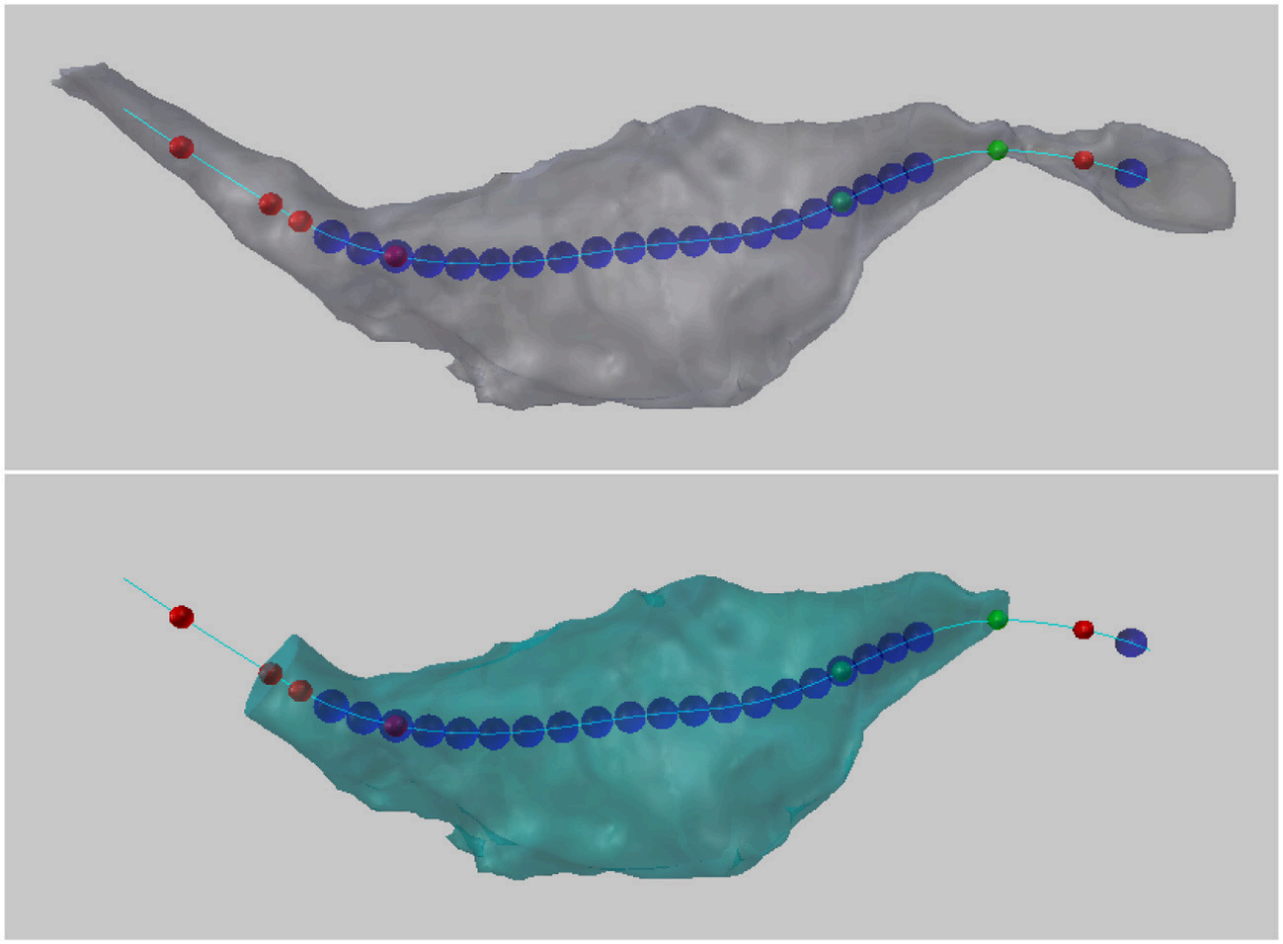
**(Top)** Colored balls mark certain geometric criteria that potentially define a bouton. In this example, the blue spheres indicate the position along the centerline where the maximum radius of the cross-sectional surface area was greater than 0.2 micrometers. Red spheres indicate where the cross sectional surface area of the axon is decreasing by a factor of 2 over a distance 0.2 micrometers (looking from right to left). Green spheres indicate where the cross sectional surface area of the axon is increasing by a factor of 2 over a distance 0.2 micrometers (looking from right to left). **(Bottom)** Two user-chosen balls are used to define and extract a new bouton object.

Colored spheres are then placed along the centerline at locations that meet certain geometric constraints based on these values, as follows, see Figure 13.

#### 5.6.1. Increasing/decreasing cross-sectional surface area

If the cross-sectional surface area is increasing or decreasing by at least a factor of *A* (*A* = 2.0 in Figure 13) over a distance of *D* (*D* = 0.2 Figure 13) along the axon, this might indicate the presence of a bouton. (The direction of increase is defined from the lowest to the highest centerline vertex index used in its mesh representation in Blender.)

- A *green sphere* indicates that somewhere over the next *D* distance along the centerline, there is a vertex whose cross-sectional surface area is at least *A* times larger than the surface area at the vertex with the green sphere. If there are two green spheres in a row, this condition is true for each of them independently; the spheres do not mark the entire region of area increase, they only mark where the condition starts.
- A *red sphere* indicates that somewhere over the next *D* distance along the centerline, there is a vertex whose cross-sectional surface area is at least *A* times smaller.

#### 5.6.2. Large cross-sectional radius

If the maximum radius of the cross section at a vertex is greater than *M* (*M* = 0.2 in Figure 13), this might indicate the presences of a bouton. The radius is measured from the centroid of the cross section (the average location of all its vertices) to each of its vertices separately, and the maximum radius is defined as the largest of these distances.

- A *blue sphere* indicates that the maximum radius of the cross section at that vertex is greater than *M*.

From the possibly many spheres placed along the centerline, the user can select the two that they decide best bound the desired bouton. The tool will then extract the region of the axon between these two points, returning a new bouton object whose volume can be calculated using the NeuroMorph Measurement tools as described in section 4.

## 6. Proximity analysis

The tools described in this section enable the analysis of regions of two different classes of objects that are close to one another.

### 6.1. Sphere to surface distances

**Figure 14:**
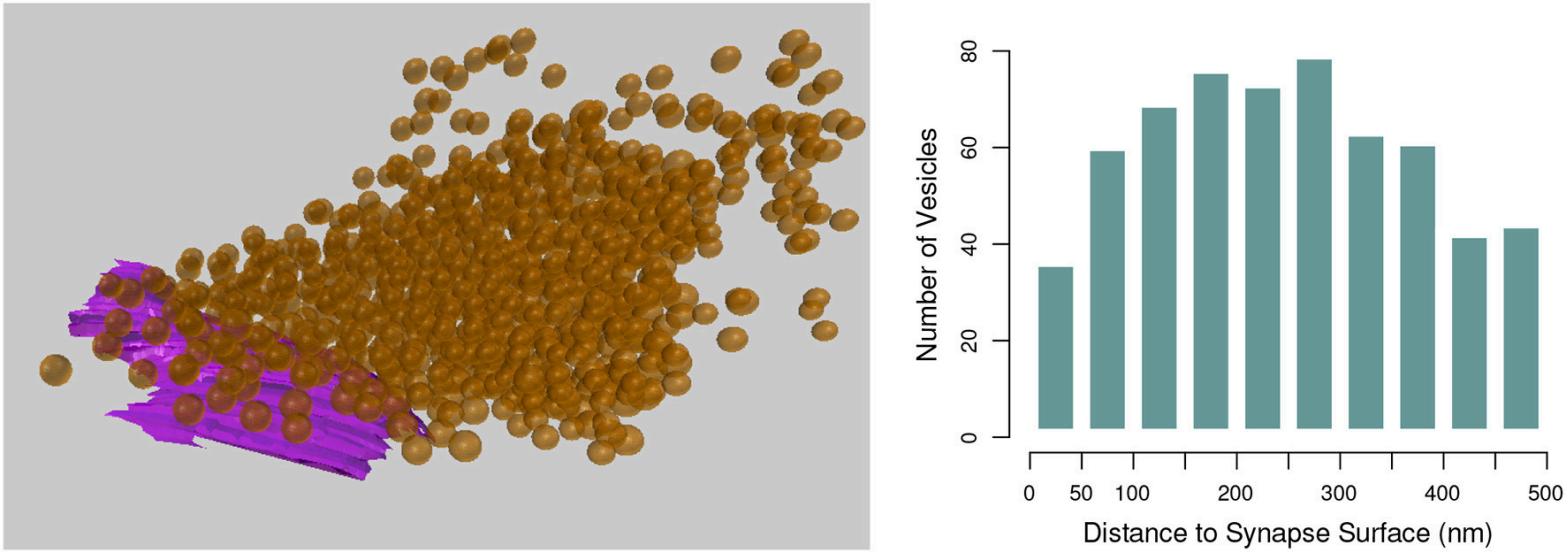
**(Left)** The shortest distance from each vesicle sphere center (orange) to the pre-synaptic surface (purple) is calculated. **(Right)** A histogram showing the numbers of vesicles in each 50 nm bin from the pre-synaptic surface.

This tool computes the shortest distance in 3D from each instance of one class of object, such as the vesicles created in section 3.1, to a given mesh object, such as a synapse surface created in section 3.2, see Figure 14. This tool was first developed for use in Barnes et al. (2015).

Each vesicle object is assumed to be a sphere, and only their centroids are used in the calculation. Non-spherical mesh objects will be processed without question, but the user should keep in mind that irregularly shaped objects may not be well-represented by the centroid of their vertices. The distance from the centroid of each vesicle to each vertex on the selected synapse surface is calculated, and the shortest distance found for each vesicle is exported in an output file.

### 6.2. Interacting regions

**Figure 15:**
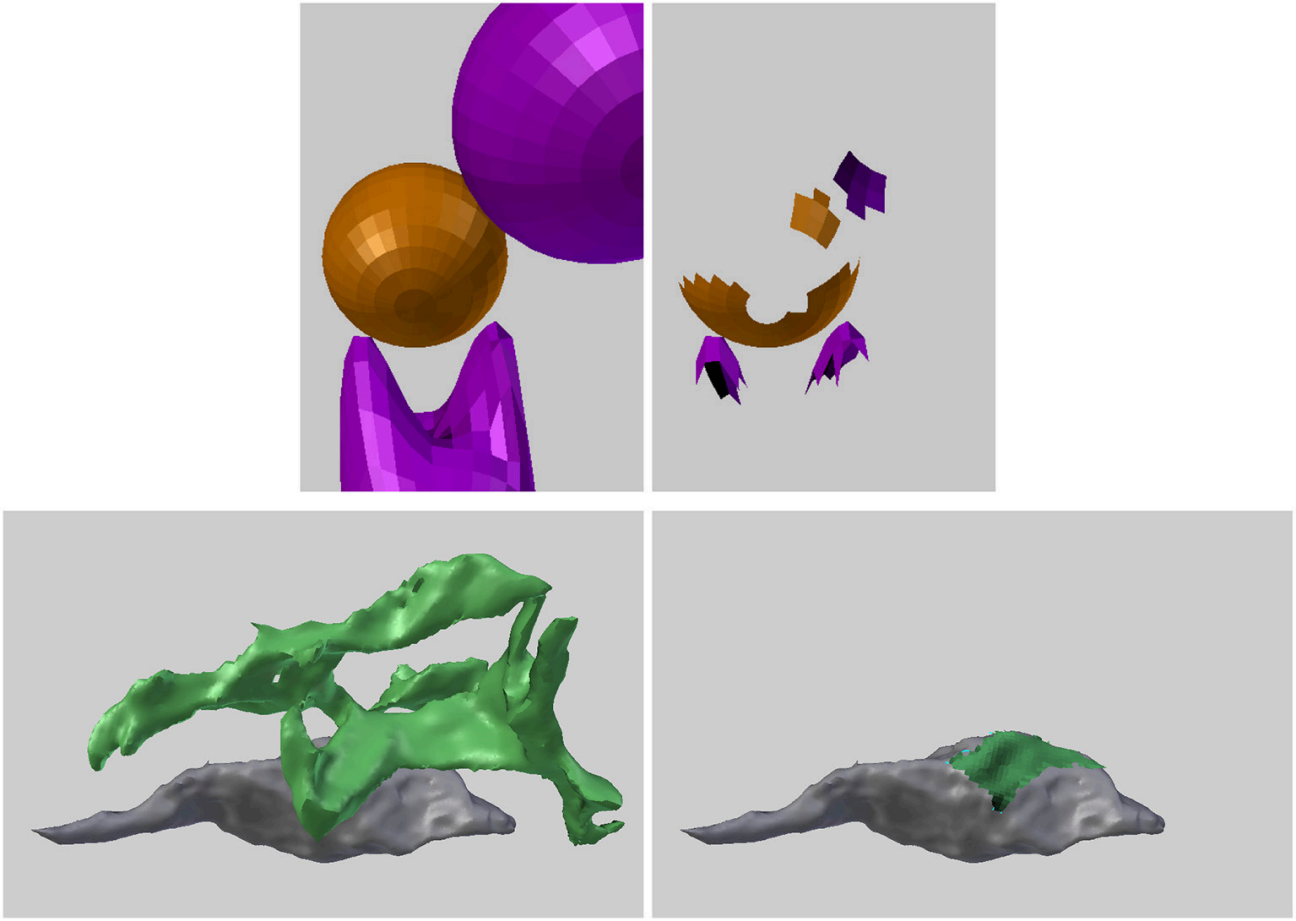
**(Upper left)** Two different types of objects (orange and purple) are close to each other in space. **(Upper right)** The regions of the two object types that are less than a given distance apart are identified and extracted. The potential physical interactions of nearby biological structures are often important, and there are many biological applications of this functionality. **(Lower left)** Part of a green astrocytic process lies close to an gray axonal bouton. **(Lower right)** The proximity tool was used to extract and measure the area of astrocytic process (green) within 50 nm of the bouton.

The NeuroMorph toolset also provides functionality to extract the regions of two different objects that lie within a certain distance of each other. Here, we show in Figure 15 how it can be used to measure the area of apposition between the axonal bouton and an astrocytic process that lies alongside. The functionality could equally be used to analyze the interactions between endoplasmic reticulum and mitochondria.

Given two different objects, or two different classes of objects with pieces joined together into two Blender objects, the Proximity Analysis tool extracts the regions of the objects that are less than a user-defined distance threshold *T* from each other, see Figure 15, and exports the corresponding surface area pairs of each interaction sub-region for analysis.

All interacting sub-regions of the two input objects will be created as child objects of the original input objects. The objects are initially not visible, in order to not clutter the scene, but can be viewed individually using Blender's visibility toggle. All child objects can be made visible together using the NeuroMorph Parent-Child Tools, provided in NeuroMorph's “Other Tools” toolbox.

The algorithm works by determining all vertices on each object that are less than the distance *T* from a vertex in the other object. A k-d tree (Bentley, 1975), which is an optimized geometric search structure, is used to speed up the processing time of the distance calculations. The vertices are grouped into contiguous units on each object, and paired with their corresponding nearby regions on the other object. The surface areas of each of these distinct sub-regions are calculated.

The output file provides the names of the sub-regions of the first object (e.g., “object1.001”), their surface areas, the corresponding regions of the second object (e.g., “object2.027”), their surface areas, and the centroid of the two regions in order to provide some context for the interaction in space. It also provides the total non-overlapping surface area of each object class, which is generally not the same as the sum of the surface areas of each individual sub-region, see below.

#### 6.2.1. Understanding the output

##### 6.2.1.1. Regions where surface area = 0

Edges and vertices that are not part of any faces are cleaned away at the end of the procedure. This means that there may be a region of mesh faces on one object with no corresponding region on the second object, because the corresponding region consisted only of vertices or edges, but no full faces, so had a surface area of 0. If these deleted regions are important to the user, a finer mesh should be provided where entire faces lie within the threshold distance. Remeshing, or simply subdividing faces to result in a finer mesh, is a straightforward operation in Blender^14^.

##### 6.2.1.2. Doubly counted overlapping regions

The provided results consist of pairs of interacting individual contiguous mesh regions from each object. If a region on one object corresponds to two separate regions on the second object, its surface area will be included in two separate entries in the output file. For this reason, summing the interacting surface areas may result in a greater overall surface area than the true area in space. This may be the desired result, depending on the application. The last line of the output file provides the total non-overlapping surface area for each object.

## 7. Discussion

Serial electron microscopy is now a commonplace technique for exploring cell and tissue structure. Although many methods have appeared in recent years for reconstructing different features from the image stacks (e.g., Morales et al., 2011; Sommer et al., 2011; Cardona et al., 2012; Belevich et al., 2016), few provide any means by which geometric data can be extracted directly from the resulting 3D models. The NeuroMorph tools were primarily constructed, therefore, not as a tool for segmentation or reconstruction, but to allow the user to make detailed measurements of any part of the models. We have integrated these into the Blender software as this open-source platform is arguably the most comprehensive and well-maintained of its type. Its 3D view allows the user to manipulate any part of a mesh while simultaneously viewing the original bitmap images, therefore, providing the opportunity to further add to the models or make corrections.

Blender's versatility as modeling and visualization software has been exploited for other biological applications, leading to the creation of independent tools developed for this platform. BioBlender (Andrei et al., 2012) was developed as a molecule visualization tool so that molecular models imported from various sources can be viewed, and manipulated. This enables their physical and chemical properties to be included so that their activity can be seen in a realistic way. Similarly, MCell (Kerr et al., 2008) is a Blender-based piece of software into which cellular models can be imported and populated with different molecules that are assigned with their kinetic properties. The software can then carry out particle-based Monte Carlo simulations to understand the molecular diffusion and interactions within biologically relevant cellular geometries.

The NeuroMorph toolset is entirely complementary to these other software packages, giving the user the ability to quantify the geometry. We show here how the different parts of the software can provide details about the morphology from a single glutamatergic axon, but all of this functionality could equally be used to study any other cellular elements represented by mesh models. The computational functionality of NeuroMorph is limited only by the speed and memory of the computer on which it is run. We have successfully tested scenes with many hundreds or thousands of objects on a personal computer. When only the location of an object matters, NeuroMorph also provides a tool to reduce objects to their centroids, so that more objects can be processed.

As the list of algorithms for segmenting different features from serial electron microscopy images grows, tools such as these will become increasingly more in demand as scientists continue to map and quantify cellular environments.

## Author contributions

AJ and GK conceived and planned the development of this software. AJ developed the software which GK and JB tested. JB constructed the models shown and provided with this paper. AJ and GK wrote the paper. GK supervised the project.

### Conflict of interest statement

The authors declare that the research was conducted in the absence of any commercial or financial relationships that could be construed as a potential conflict of interest.

## Footnotes

^1^fiji.sc

^2^knossostool.org

^3^cajalbbp.cesvima.upm.es/espina

^4^synapseweb.clm.utexas.edu/software-0

^5^http://www.itksnap.org/pmwiki/pmwiki.php

^6^www.fei.com/software/amira-avizo

^7^neuromorph.epfl.ch

^8^https://github.com/NeuroMorph-EPFL/NeuroMorph

^9^mcell.org

^10^www.blender.org

^11^https://fiji.sc/

^12^http://mib.helsinki.fi

^13^http://www.vmtk.org/tutorials/Centerlines.html

^14^https://docs.blender.org/manual/en/dev/modeling/modifiers/generate/remesh.html

## References

[B1] Andrei R. M. Callieri M. Zini M. F. Loni T. Maraziti G. Pan M. C.. (2012). Intuitive representation of surface properties of biomolecules using BioBlender. BMC Bioinformatics 13(Suppl 4):S16. 10.1186/1471-2105-13-S4-S16 22536962 PMC3434447

[B2] Antiga L. Piccinelli M. Botti L. Ene-Iordache B. Remuzzi A. Steinman D. A. (2008). An image-based modeling framework for patient-specific computational hemodynamics. Med. Biol. Eng. Comput. 46:1097. 10.1007/s11517-008-0420-1 19002516

[B3] Antiga L. Steinman D. A. (2004). Robust and objective decomposition and mapping of bifurcating vessels. IEEE Trans. Med. Imaging 23, 704–713. 10.1109/TMI.2004.826946 15191145

[B4] Barnes S. Cheetham C. E. J. Liu Y. Bennett S. Albieri G. Jorstad A. A. (2015). Delayed and temporally imprecise neurotransmission in reorganizing cortical microcircuits. J. Neurosci. 24, 9024–9037. 10.1523/JNEUROSCI.4583-14.2015 PMC4469734 26085628

[B5] Belevich I. Joensuu M. Kumar D. Vihinen H. Jokitalo E. (2016). Microscopy image browser: a platform for segmentation and analysis of multidimensional datasets. PLoS Biol. 14:e1002340. 10.1371/journal.pbio.1002340 26727152 PMC4699692

[B6] Bentley J. L. (1975). Multidimensional binary search trees used for associative searching. Commun. ACM 18, 509–517. 10.1145/361002.361007

[B7] Briggman K. L. Denk W. (2006). Towards neural circuit reconstruction with volume electron microscopy techniques. Curr. Opin. Neurobiol. 16, 562–570. 10.1016/j.conb.2006.08.010 16962767

[B8] Cardona A. Saalfeld S. Schindelin J. Arganda-Carreras I. Preibisch S. Longair M.. (2012). TrakEM2 software for neural circuit reconstruction. PLoS ONE 7:e38011. 10.1371/journal.pone.0038011 22723842 PMC3378562

[B9] Fiala J. C. (2005). Reconstruct: a free editor for serial section microscopy. J. Microsc. 218, 52–61. 10.1111/j.1365-2818.2005.01466.x 15817063

[B10] Gala R. Lebrecht D. Sahlender D. A. Jorstad A. Knott G. Holtmaat A.. (2017). Computer assisted detection of axonal bouton structural plasticity in *in vivo* time-lapse images. eLife 6:e29315. 10.7554/eLife.29315 29058678 PMC5675596

[B11] Helmstaedter M. Briggman K. L. Denk W. (2011). High-accuracy neurite reconstruction for high-throughput neuroanatomy. Nat. Neurosci. 14, 1081–1088. 10.1038/nn.2868 21743472

[B12] Jorstad A. Nigro B. Cali C. Wawrzyniak M. Fua P. Knott G. (2015). Neuromorph: a toolset for the morphometric analysis and visualization of 3d models derived from electron microscopy image stacks. Neuroinformatics 13, 83–92. 10.1007/s12021-014-9242-5 25240318 PMC4303738

[B13] Kerr R. A. Bartol T. M. Kaminsky B. Dittrich M. Chang J.-C. J. Baden S. B.. (2008). Fast Monte Carlo simulation methods for biological reaction-diffusion systems in solution and on surfaces. SIAM J. Sci. Comput. 30, 3126–3149. 10.1137/070692017 20151023 PMC2819163

[B14] Kornfeld J. Denk W. (2018). Progress and remaining challenges in high-throughput volume electron microscopy. Curr. Opin. Neurobiol. 50, 261–267. 10.1016/j.conb.2018.04.030 29758457

[B15] Morales J. Alonso-Nanclares L. Rodríguez J.-R. DeFelipe J. Rodríguez Á. Merchán-Pérez Á. (2011). Espina: a tool for the automated segmentation and counting of synapses in large stacks of electron microscopy images. Front. Neuroanat. 5:18. 10.3389/fnana.2011.00018 21633491 PMC3099746

[B16] Sommer C. Straehle C. Köthe U. Hamprecht F. A. (2011). Ilastik: interactive learning and segmentation toolkit, in 2011 IEEE International Symposium on Biomedical Imaging: From Nano to Macro (Chicago, IL), 230–233.

[B17] Yushkevich P. A. Piven J. Hazlett H. C. Smith R. G. Ho S. Gee J. C.. (2006). User-guided 3D active contour segmentation of anatomical structures: significantly improved efficiency and reliability. NeuroImage 31, 1116–1128. 10.1016/j.neuroimage.2006.01.015 16545965

